# The interactions of astrocytes and fibroblasts with defined pore structures in static and perfusion cultures

**DOI:** 10.1016/j.biomaterials.2010.11.046

**Published:** 2011-03

**Authors:** Tao Sun, Peter S. Donoghue, Jennifer R. Higginson, Nikolaj Gadegaard, Susan C. Barnett, Mathis O. Riehle

**Affiliations:** a Centre for Cell Engineering, Institute of Molecular, Cellular and Systems Biology, College of Medical, Veterinary and Life Sciences, Joseph Black Building, University of Glasgow, Glasgow G12 8QQ, UK; b Institute of Infection, Immunity and Inflammation, College of Medical, Veterinary and Life Sciences, Glasgow Biological Research Centre, University of Glasgow, Glasgow G12 8TA, UK; c Division of Biomedical Engineering, School of Engineering, University of Glasgow, Glasgow G12 8LT, UK

**Keywords:** Scaffold porosity, Fibroblast, Astrocyte, Static culture, Perfusion culture, Tissue engineering

## Abstract

Open pores to maintain nutrient diffusion and waste removal after cell colonization are crucial for the successful application of constructs based on assembled membranes, in our case tubular scaffolds made of ɛ-polycaprolactone (PCL), for use in tissue engineering. Due to the complex three-dimensional structure and large size of such scaffolds needed for transplantable tissues, it is difficult to investigate the cell–pore interactions *in situ*. Therefore miniaturized bioreactors inside Petri dishes (30 mm in diameter), containing porous PCL or poly-dimethylsiloxane (PDMS) membranes, were developed to allow the interactions of different cells with defined pores to be investigated *in situ* during both static and perfusion cultures. Investigation of two different cell types (fibroblasts and cortical astrocytes) and how they interact with a range of pores (100–350 μm in diameter) for up to 50 days indicated that the cells either ‘covered’ or ‘bridged’ the pores. Three distinct behaviors were observed in the way cortical astrocytes interacted with pores, while fibroblasts were able to quickly bridge the pores based on consistent “joint efforts”. Our studies demonstrate that the distinct pore sealing behaviors of both cell types were influenced by pore size, initial cell density and culture period, but not by medium perfusion within the range of shear forces investigated. These findings form important basic data about the usability of pores within scaffolds that could inform the design and fabrication of suitable scaffolds for various applications in tissue engineering.

## 1 Introduction

The extracellular matrix (ECM) plays an important role in controlling cell behaviors *in vivo*. Accordingly, considerable research has been undertaken to mimic three-dimensional (3D) ECM for tissue engineering scaffolds by generating well-defined architectures including porosity, surface topography and chemistry for optimizing cell and tissue growth [1–3]. It is well documented that cells have various sensitivity length scales [4–6] and respond to local macro-, micro-, and nano-scale patterns of chemistry and topography differently [2]. For example, if the topography is below their minimum sensing scale, cells will mainly respond to chemistry [7]. Within a certain range, topographic cues can influence cell behavior independent of the underlying material chemistry [5,8,9]. If structures are appreciably wider than cells, the topographic effects will diminish dramatically [6,10].

Previously by combining prior knowledge of cell responses to chemical [9], micro- and nano- topographical cues [10,11], we have fabricated tubular constructs (coined ‘Swiss roll’) with potential applications in vascular and nerve tissue engineering [12,13]. The Swiss rolls were made of a 15–50 μm thick biodegradable ɛ-polycaprolactone (PCL) [14] membrane with micro- and/or nanotopographies on either (both) side(s) to guide/promote or inhibit specific cellular responses. In addition to the topographic cues, open pore structures on the PCL membrane to increase scaffold porosity are crucially important, as the PCL membrane is not permeable and nutrient/waste diffusion through both ends of the >5 mm long tubular construct is very limited. In order to develop an engineered 3D scaffold with optimal pore size and the highest possible porosity without compromising its mechanical strength, it is important to systematically investigate the influence of pore structures on cell behavior.

The success of tissue engineering is dependent not only on the migration of cells within the scaffolds but also on their survival by the maintenance of nutrient diffusion throughout the scaffold after cell colonization [15,16]. For the culture of metabolically demanding tissues, it is likely that perfusion culture will better support metabolic activity rather than changing the medium every few days [17,18] due to the limited mass transport between bulk medium and the cells within the majority of reconstructed tissues [19]. Therefore, although the interactions of cells with defined pore features have been studied previously [15,20,21], it is necessary to systematically investigate different cell types on porous substrates not only in static culture but also under perfused conditions. Due to the tube like shape, the large size, the internal micro-topographies of the 3D scaffolds, and the translucency of the PCL membrane, it is almost impossible to observe living cells within the Swiss rolls during static or perfusion culture using currently available techniques. Thus we have developed miniaturized static and perfusion culture systems incorporated into small Petri dishes (30 mm in diameter). In each of the Petri dish-based mini-bioreactors, a cell-loaded thin PCL or poly-dimethylsiloxane (PDMS) membrane with defined pore features was suspended in the medium, and cells within the pores could be observed in real time using a variety of microscopic techniques during static, or perfusion culture. Thus, the mini-bioreactors can be used as *in vitro* simulators to mimic the internal configuration of the membrane based porous 3D scaffolds and predict the cellular behaviors within, which is crucial for the optimization of scaffold design [15,20,21].

In order to increase the generality and transferability of this research, and also because of the potential applications of the Swiss rolls to various areas of tissue engineering, two different cell types were selected in this research. As the major supportive glial cell in the central nervous system [22,23], type 1 cortical astrocytes were selected as an exemplary cell model that would encounter structures in nerve tissue engineering. hTERT fibroblasts were selected because fibroblasts form extracellular matrix in many tissues and play important roles in various wound healing processes [24,25].

## 2 Materials and methods

### 2.1 Cell culture

As described previously [22,26] purified type 1 astrocytes were prepared by first digesting cortices (dissected from 1 day old Sprague Dawley rats) in 1.33% collagenase (Sigma, Poole, UK), seeding (∼2 × 10^7^ cells per T75 flask) and culturing the cells in poly-l-lysine coated T75 flask for 10–12 days. The cells were maintained in DMEM (Invitrogen, Paisley, Scotland) supplemented with 10% fetal bovine serum (FBS) (Invitrogen, Paisley, Scotland) and l-glutamine (2 mm, Sigma). Confluent flasks were shaken on a rotary platform overnight at 37 °C to remove contaminating oligodendrocyte precursor cells. The remaining cells after this procedure were 85–95% type 1 astrocytes as judged by labeling for glial fibrillary acidic protein (GFAP), a cell type specific marker for astrocytes. The other 5–15% were mainly oligodendrocyte precursor cells and type 2 astrocytes, which were identified with immuno-fluorescent microscopy in preliminary experiments (shown as supplementary data). These cells had bi-, tri- or multi- polar morphologies and were apparent only at an early culture stage (4–5 days). After that, type 1 astrocytes gradually dominated the culture. The astrocytes were passaged no more than 4 times and detached using trypsin/EDTA (0.02% solution) for experiments when almost 100% confluent. hTERT fibroblasts (immortalised from primary human BJ foreskin fibroblasts, Clontech Laboratories, Inc. USA) were cultured using the same medium as astrocytes and detached for experiments when approximately 90% confluent.

### 2.2 Fabrication and treatment of PDMS and PCL membranes with defined pores

The poly-dimethylsiloxane (PDMS) membranes with defined pores were prepared through a number of steps. Initially, silicon wafers with micro-pillars of different sizes (Fixed height: 100 μm, various diameters: 100, 150, 180, 280, 300, 350 μm) were fabricated as previously reported [27]. PDMS membranes (approximately 100 μm thick) were made by spin coating Sylgard 184 (Dow Corning, Bad Homburg, Germany) at 10: 1 (prepolymer: curing agent) on the silicone wafers. After curing for 2–3 h at 80 °C, cooled down to room temperature, the porous PDMS sheet was carefully peeled off from the silicon wafer, trimmed into smaller square pieces (15 mm × 15 mm), framed by gluing four sides onto thin plastics using Sylgard 184 at 10 : 1 (prepolymer: curing agent) and again cured for 2–3 h at 80 °C. Porous ɛ-polycaprolactone (PCL, Mw 65000, CAS 24980-41-4, Aldrich, Poole, UK) membranes with fixed pore size (300 μm in diameter) were prepared using a slightly simpler procedure. Briefly, very thin PCL membranes were made by spin coating 25% (w/v) PCL in chloroform solution onto the silicon wafers with micro-pillars (Fixed height: 10 μm, diameter: 300 μm) as previously described [27]. After the chloroform had completely evaporated, the PCL membrane was peeled off from the silicon wafer, and trimmed into smaller square pieces (15 mm × 15 mm) for cell culture.

Prior to experimentation both the PDMS and PCL membranes were sterilized with 70% ethanol for 24 h, rinsed thoroughly with sterilized RO water, dried, treated with a Harrick Plasma Cleaner (Harrick Plasma, USA) at Hi settings (740 V DC, 40 mA DC, 29.6 W) for 1 and 5 min respectively, coated with collagen I solution (APCOLL-S, Purified Soluble Collagen (3.1 mg/ml), a gift from Devro Medical, Glasgow, UK) for 15 min and then washed thoroughly with phosphate-buffered saline (PBS). After that the membranes were fixed in 6-well tissue culture plates with a silicone ring (outside diameter 30 mm, inside diameter 15 mm), seeded with 2 ml of cell suspension (approximately 1 × 10^5^ cells/ml). After static incubation (37 °C and 5% CO_2_) for 2 h to allow full cell attachment on the upper side (cell seeding side), the cell-loaded membranes were incorporated into one of the mini-bioreactors as described in the next section.

### 2.3 Design and fabrication of miniaturized static and perfusion culture modules

**Fig. 1:**
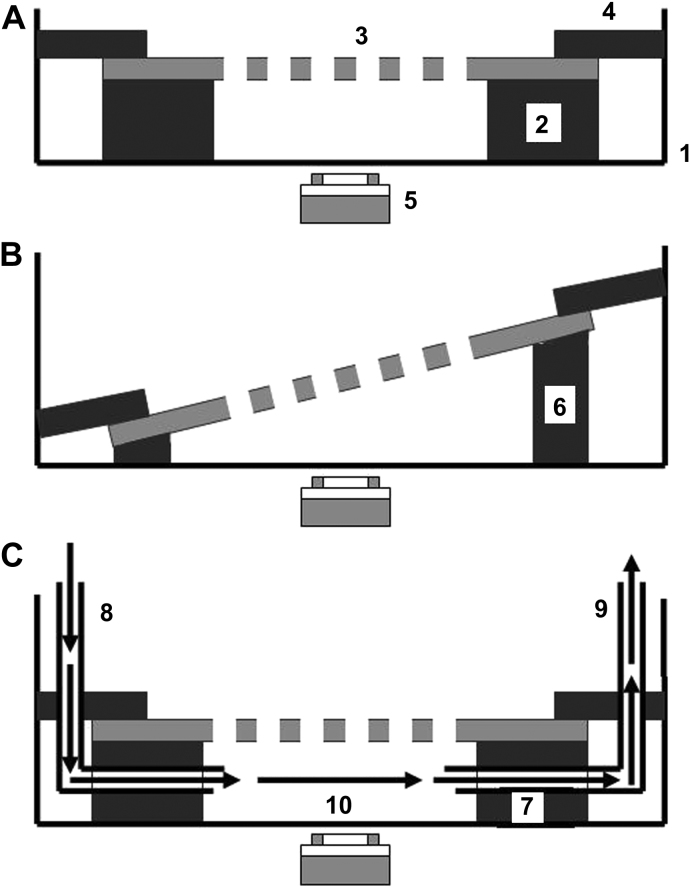
Schematic diagrams of static culture modules (A) I, (B) II, and (C) the perfusion culture module. In static culture module I: (1) a small Petri dish (3 cm inside diameter) was used as the cell culture chamber, inside which was a sandwich starting at the bottom with (2) a silicone O-ring spacer (outside diameter 30 mm, inside diameter 15 mm, thickness: 1 mm), (3) a piece of cell-loaded porous substrate, and (4) a plastic O-ring (outside diameter: 30 mm, inside diameter: 20 mm) to fix the porous membrane in place. Medium (2–3 ml) was added into the chamber to make sure the cell-loaded membrane was exposed to the medium on both sides. During culture the cells on the substrate were monitored non-invasively using (5) a microscope. In static culture module II: (6) a silicone O-ring spacer with gradient thickness (outside diameter: 30 mm, inside diameter: 15 mm, gradient thickness from the thin side to the thick side: 0.5–3 mm) was used such, that the cell-loaded porous substrate was tilted in the medium. In the perfusion culture module: (7) a circular silicone spacer (30 mm in diameter, 1 mm thick) with a rectangle chamber (5 mm × 25 mm) created inside was used, two L-shape cannulae (18 G1^1^/_2_″) were inserted through a plastic O-ring and the bottom silicone spacer into the rectangular perfusion chamber made up by the cell-loaded porous substrate, the silicone spacer and the bottom surface of the Petri dish were used as (8) the inlet and (9) the outlet respectively. The rectangular chamber was subjected to (10) medium perfusion with varying flow rates.

In order to investigate the interactions of cells with defined pores from different perspectives in static and perfusion cultures, two static culture modules and one perfusion culture module were designed and fabricated. As illustrated in Fig. 1A–C, small Petri dishes (inside diameter 30 mm) were used as the culture chambers for all the modules. Inside the chamber of static culture module I (Fig. 1A) was a sandwich starting at the bottom with a piece of silicone O-ring spacer (outside diameter 30 mm, inside diameter 15 mm, thickness: 1 mm), a piece of cell-loaded PCL or PDMS membrane with defined pores, and a plastic o-ring (outside diameter 30 mm, inside diameter 20 mm) to fix the cell-loaded membrane in position. Approximately 2–3 ml of medium was added into the chamber to make sure the cell-loaded membrane was exposed to the medium on both sides. The same concept was used in static culture module II (Fig. 1B), however a silicone O-ring spacer with gradient thickness (outside diameter 30 mm, inside diameter 15 mm, gradient thickness from the thin side to the thick side: 0.5–3 mm) was used, thus the cell-loaded porous membrane was tilted in the medium. There are two advantages of this design compared with static module I: (1) the complex cell structures inside the pores can be investigated optically from different angles, (2) the exchange of cells between two sides of the membrane through the pores can be observed directly. In the perfusion culture module (Fig. 1C), a circular silicone spacer (30 mm in diameter, 1 mm thick) with a rectangular chamber (5 mm × 25 mm) created inside was used. Two L-shape cannulae (18 G1^1^/_2_″) were inserted through the plastic O-ring and the bottom silicone spacer into the rectangular perfusion chamber (made up by the cell-loaded porous membrane, the silicone spacer and the bottom surface of the Petri dish), and used as inlet and outlet respectively. The cannulae were connected with an Ismatec peristaltic pump (IPC-N, ISMATEC, Switzerland), a medium reservoir (2.5 ml) and a 3-way valve by silicone tubes (Inside diameter: 1 mm). The rectangular chamber was subjected to medium perfusion with varying flow/shear rates (see Table 1).

Prior to assembly, all the components of the mini-bioreactors (except the cell-loaded porous membranes) were sterilized in 70% (v/v) ethanol/30% (v/v) sterile water overnight, then washed thoroughly with PBS, sterilized RO water and dried. After the cell-loaded porous membranes were incorporated, all the chambers were filled with 2–3 ml of medium and placed in a larger sterilized Petri dish (9 cm in diameter) filled with 95% air/5% CO_2_ at 37 °C for subsequent static or perfusion cultures. During culture, the cells in all the modules were monitored consistently using various non-invasive microscopic techniques including phase contrast time-lapse microscopy, live cell fluorescent microscopy and scanning ion conductance microscopy (SICM) [28]. After culture, all the modules were disassembled and all the components apart for the PCL and PDMS substrates were reusable after thorough washing and sterilization with 70% (v/v) ethanol.

### 2.4 Live cell fluorescence microscopy

Cells cultured in the modules were also analysed using live cell fluorescent microscopy. Briefly, MitoTracker^®^ Red 580 (Molecular Probes, Invitrogen) stock solution (1 mm) was added into the culture medium in the module chamber to a final concentration of 100 nm. After 45 min incubation the cells were imaged using a fluorescence microscope.

## 3 Results

### 3.1 Astrocyte cultures on porous PCL membranes in static culture module I

**Fig. 2:**
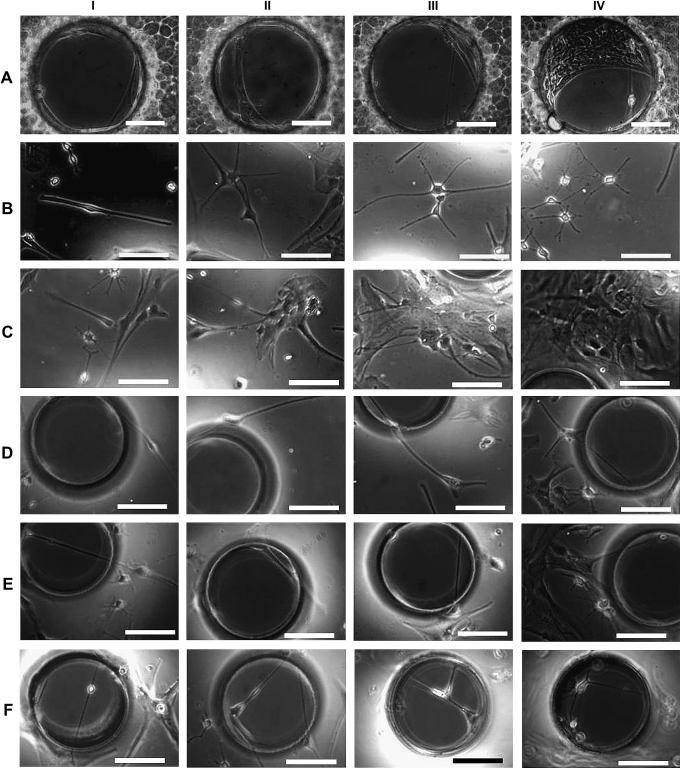
Phase contrast micrographs of cells in cortical astrocyte cultures (A) within the pores of porous polycaprolactone (PCL) membranes after having been cultured for more than 4 days in static culture module I. Phase contrast micrographs of cells in cortical astrocyte cultures on (B and C) non-pore areas, (D and E) close to the pore edges and within the pores of porous poly-dimethylsiloxane (PDMS) membranes after 2–4 days in the static culture module I. Bar = 100 μm.

Cortical astrocytes were loaded onto PCL membranes with defined pores (diameter: 300 μm) in static culture module I and monitored using time-lapse phase contrast microscope. Due to the translucent nature of the PCL membrane, cells on the non-pore areas were hard to observe, thus only the cells within the pores could be followed. Elongated single cells, large membranes of multiple cells or combination of both were found crossing 10–20% of the pores within 4 days culture as shown in Fig. 2A. Instead of remaining in the pores statically, these cell structures dynamically slid forward and backward across the pores. Most of the elongated single cells pulled apart eventually due to further stretching across the pores, whereas the large cell made membranes persisted and either slowly migrated away, or were pulled out of the pores after further culture for 10–14 days, which suggested the necessity to also observe cell behaviors on non-pore areas.

### 3.2 Astrocyte cultures on porous PDMS membranes in static culture modules I and II

**Fig. 3:**
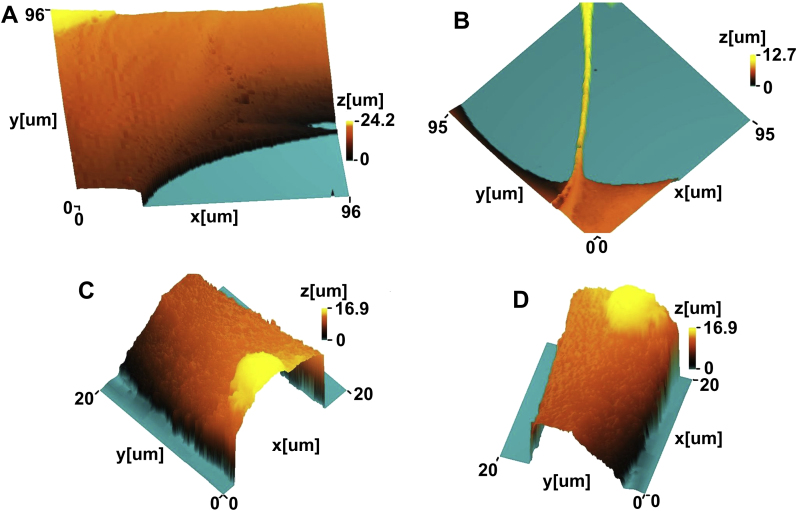
Micrographs of the cells in cortical astrocyte cultures imaged *in situ* using scanning ion conductance microscopy (SICM) during cell culture (A) on the pore edge, (B) partly on the pore edge, partly in the pore, and (C and D) in the pores of porous poly-dimethylsiloxane (PDMS) membranes in static culture modules I.

In order to observe cells both on non-pore areas and within the pores, astrocytes were cultured on PDMS membranes with defined pore features in static culture modules I and II, monitored using time-lapse microscopy. Due to the transparency of PDMS membranes, the cells on the non-pore areas (Fig. 2B–C), close to the edges of the pores and inside the pores (Fig. 2D–F) could be observed and tracked continuously. Morphological and topographical analysis of these cell structures both on the edges (Fig. 3A and B), and inside the pores (Fig. 3B–D) in real time using SICM indicated that the elongated cells were as thin as 5–10 μm, while the large membranes were almost the same sizes as the pores (100–350 μm).

**Fig. 4:**
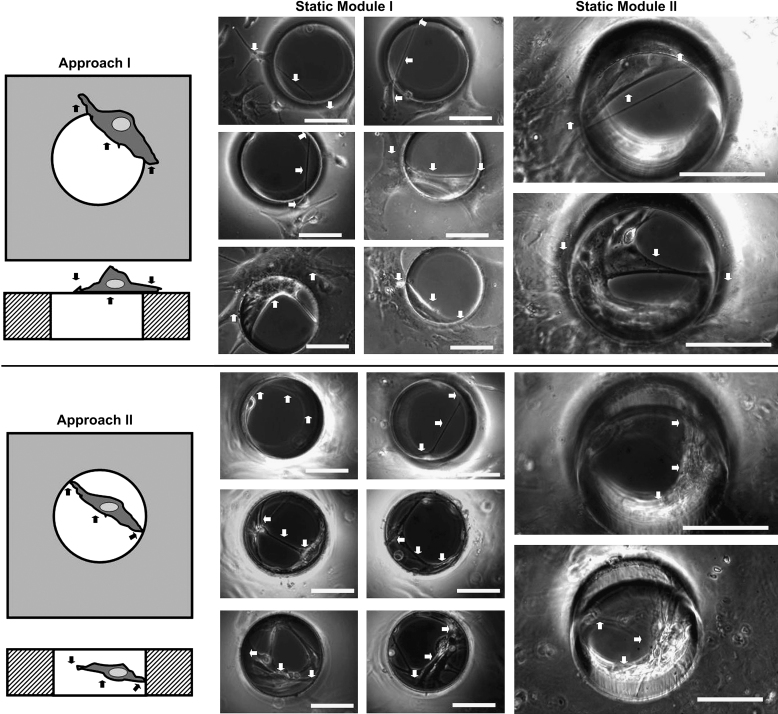
Schematic diagrams of the two approaches used by different cells in cortical astrocyte culture to interact with the pores on PDMS membranes and corresponding example phase contrast micrographs obtained from static culture modules I and II. Approach I: cell(s) ‘covered’ the pores without migrating inside. Approach II: Cell(s) ‘bridged’ the pores by migrating inside and attaching onto the inner aspects of the pores. The cell structures covered the pores (Approach I) or were suspended within the pores (approach II), their attachment points on non-porous areas or onto the inner aspects of the pores are highlighted by arrows. Bar = 100 μm.

**Fig. 5:**
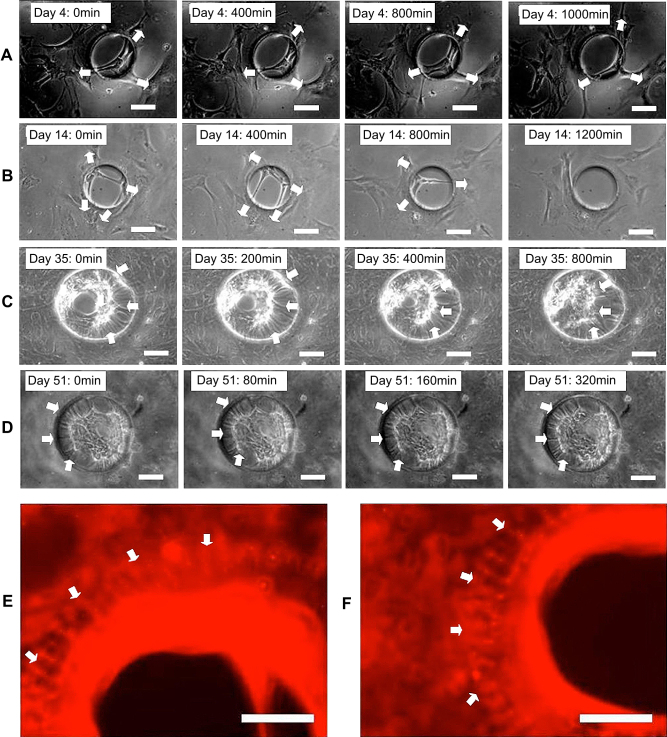
Micrographs from time-lapse videos of the cells in cortical astrocyte culture on porous PDMS membranes in static culture module I after culture for (A) 4 days and (B) 14 days indicating, that the cells within the pores were pulled out by the cells on the non-pore areas (arrows); after culture for (C) 35 days and (D) 51 days indicating the cells on the non-pore areas (arrows) were pulled towards the pores by the cells within the pores. (E, F) Fluorescence micrographs of type 1 astrocytes on porous PDMS membranes in static culture module I after culture for 30 days and stained a live with MitoTracker^®^ Red indicating the cells (arrows) around the pore were pulled towards the pores and deformed. Bar = 100 μm.

**Fig. 6:**
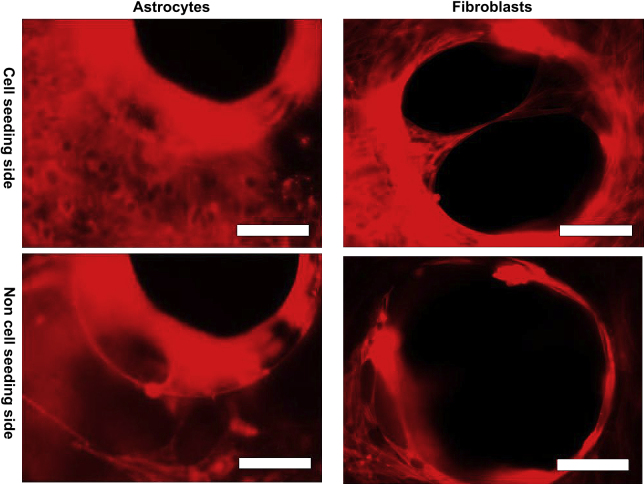
Fluorescence micrographs of cells in cortical astrocyte culture and fibroblasts on both sides of porous PDMS membranes in static culture module I after culture for 17 days and stained live with MitoTracker® Red. Bar = 100 μm.

Further investigation indicated that two different approaches were employed by cells to interact with the pores: they either ‘covered’ the pores without migrating inside (Approach I) or ‘bridged’ the pores by migrating inside and attaching onto the inner aspects of the pore (Approach II) as illustrated in Fig. 4. Long-term observation using both static culture modules found 3 distinctive cell–pore interactions at different time points during the culture period. During the first 1–4 days’ culture, individual oligodendrocyte precursor cells or type 2 astrocytes (as defined by their classic bi-, tri- or multi- polar morphologies) either ‘covered’ or ‘bridged’ the pores using both approaches as illustrated in Fig. 2D–F. After culture for 4–14 days, type 1 astrocytes gradually dominated the culture, colonizing and ‘covering’ some of the pores temporarily. However, the astrocyte colonies, that ‘covered’ the pores using Approach I, were pulled away or even torn apart/broken by other migrating astrocytes that were within the same colony but on neighboring non-porous areas as illustrated in Fig. 5A and B. After prolonged culture for more than 2–4 weeks, especially when the cultures were confluent, astrocytes were observed to gradually migrate inside the pores, attach to the pore edges, and join together forming dense circular membranes following the pore edge (Approach II). These sheets were then observed to gradually pull neighboring cells towards the pores. This action clearly deformed the cells both within the pores and those close to the pores as illustrated in Fig. 5C–F. Some cells also migrated from the cell seeding side, through the pores, onto the other side of the membranes (Fig. 6). All these sealing processes were clearly influenced by pore size as approximately 70–80% of the small holes (100 μm in diameter) were totally sealed after 2–3 weeks, while only 10–20% of the larger holes (200–350 μm in diameter) were sealed even after culturing for more than 3–4 weeks.

### 3.3 Fibroblasts on PDMS membranes with defined pores in static culture modules I and II

**Fig. 7:**
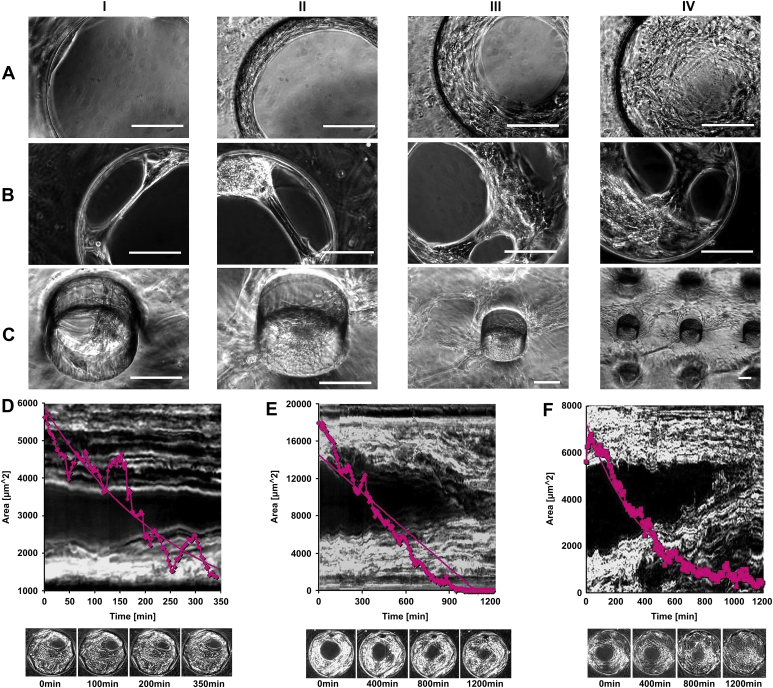
Phase contrast micrographs of fibroblasts inside the pores of porous PDMS membranes in (A, B) static culture module I and (C) static culture module II. Bar = 100 μm. Quantitative analysis using image J of the time-lapse videos of fibroblasts occluding pores after culture for (D) 7, (E) 9 and (F) 19 days on porous PDMS substrates in static culture module I. Plotted are the sizes of cell-free areas within the pores versus time, the backgrounds to the individual plots are time-matched kymographic images along the line indicated in the inset images. The dark areas in the middle were initially cell-free, but filled up over time. The insets were images taken at the time indicated.

In static culture (module I and II) individual fibroblasts were observed to migrate inside the pores, remaining attached onto the edge with both ends (Approach I), spanning the empty space whilst actively sliding across the pores by elongation and contraction. Most of the elongated single fibroblasts “ripped” due to further stretching when PDMS membranes with larger pores (>150 μm in diameter) were used for culture. The “ripped” cells were observed to immediately re-attach onto the edges of the pores starting another round of sliding. As culture progressed, more and more cells migrated into the pores, attached to each other, and/or to the pore edge and formed a circular membrane along the edge. This membrane/cell cluster gradually increased in size and sealed the pores even those ≥300 μm in diameter within 2–4 weeks culture (Fig. 7A). Quantitative analysis of three time-lapse videos with image J demonstrated that the dynamics of the pore sealing behavior was variable and did not always follow a simple linear process as shown in Fig. 7D, which confirmed our observations. Apart from circular membranes, fibroblasts were also observed to form fibers or cable like structures of different sizes that slid across the pores (Fig. 7B). As culture continued and more fiber or cable like structures joined in to form even thicker cable structures, the open pores were then sealed by these joint efforts. By combining static culture module II with time-lapse microscope, it was found that cell structures within the pores were far more complex than simple fiber and cable structures or circular membranes. The cells actually formed semi-organized complex 3D cell structures with irregular shapes, which were suspended within the pores by attaching to the insides of the pores with fibers and cables. These complex 3D cell structures were dynamic and cells could also be seen to migrate from one side of the membrane to the other, through these 3D cell structures within the pores as shown in Fig. 7C. Compared to astrocytes, fibroblasts were quicker to migrate onto the other side of the membranes through the pores as shown in Figs. 6 and 7C, and also more effective in sealing the pores as approximately 50% of the large pores (300–350 μm in diameter) were totally sealed within 2–3 weeks’ culture.

### 3.4 The influences of medium perfusion on astrocytes and fibroblasts within the pores

**Fig. 8:**
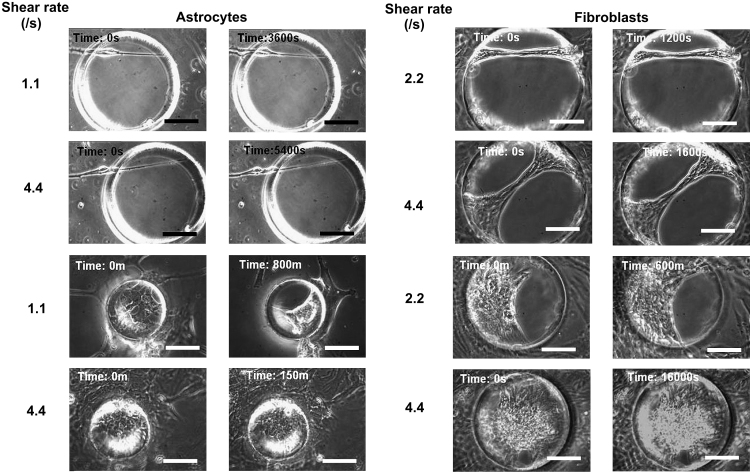
Micrographs from time-lapse videos of cells in cortical astrocyte culture and fibroblasts cultured statically on porous PDMS membranes with defined pore features in perfusion culture modules for the cells to partly or completely seal the pores, then perfused continuously from left to right (as shown in Fig. 1C) with different flow rate (80, 160, 320 ml/h) or shear rate (1.1, 2.2, 4.4/s) for different periods of times and monitored using time-lapse video microscopy. Bar = 100 μm.

Astrocytes and fibroblasts were seeded onto separate PDMS membranes with defined pores, statically cultured in the perfusion culture modules allowing cells to ‘cover’ or ‘bridge’ the pores, then perfused continuously with different flow/shear rates (see Table 1) for varying periods of time and monitored using time-lapse phase contrast microscopy. As shown in Fig. 8, although all the individual cells and/or cell membranes within the pores were observed to vibrate dramatically due to the perfusion, the sealing behaviors of both astrocytes and fibroblasts were not detectably deterred by the continuous flow of medium, even though the flow rates investigated were far beyond the optimal range for perfusion culture based on our previous experiences.

### 3.5 The influences of surface curvature on the ‘bridging behaviors’ of fibroblasts and astrocytes

**Fig. 9:**
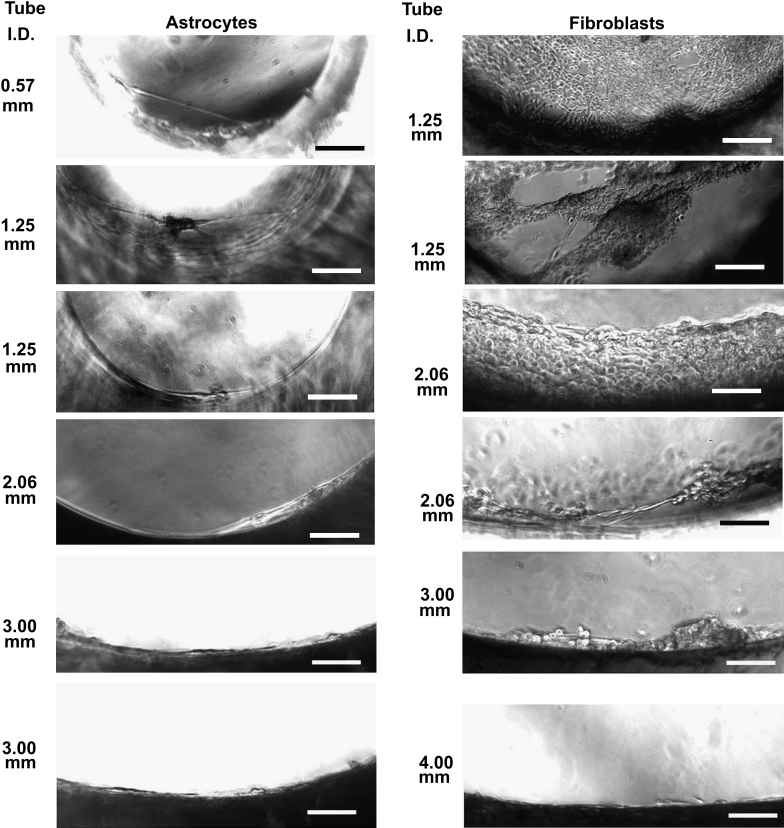
Phase contrast micrographs of cells in cortical astrocyte culture and fibroblasts after cultured inside the silicone tubes with different internal diameters (I.D.) for 14 days. Bar = 100 μm.

To further investigate their bridging behaviors, fibroblasts and astrocytes (1 × 10^5^ cells/ml) were seeded separately inside silicone tubes (treated with plasma and coated with collagen type I as described in the Materials and Methods section) with different sizes (Inside diameter (I.D.): 0.57, 1.25, 2.06, 3, 4, 5, 9 mm, length: 3 mm) and cultured for 2 weeks by keeping the silicone tubes suspended horizontally in medium. The silicone tubes were then positioned vertically in the medium for live cell imaging. The different ‘bridging’ abilities of two types of cells were observed: fibroblasts were able to ‘bridge’ up to 3 mm wide tubes while astrocytes were able to ‘bridge’ tube with a diameter up to 2.06 mm as illustrated in Fig. 9.

## 4 Discussion

Complex tissue engineering constructs based on designed membranes with micro- and nano- features, that are assembled in various 3D configurations (layers [29], or tubes [12,13,30]) have potential applications in vascular, bone, as well as peripheral and central nerve tissue engineering. Apart from micro- and nanotopographic cues to promote or inhibit various cellular behaviors, open pore features within such constructs are essential, as the successful application of such an engineered scaffold in tissue engineering is also dependent on the maintenance of nutrient diffusion throughout the scaffold after cell colonization [15]. Due to the complex tubular structure and other factors such as large size, various micro- and nanotopographies, and translucent or opaque materials, it is almost impossible to investigate the cell–pore interactions within the scaffold during culture using currently available microscopic technologies. Although other conventional cell analysis methods such as biochemical analysis, histology and immuno-staining can be used *post-hoc* at predetermined time-points, the culture has to be stopped and the cells will be sacrificed. Moreover, the fragile cell structures inside the pores will probably be damaged during the procedures such as fixation, embedding with highly viscous media, cutting and staining, thus vital information about cell–pore interactions could be lost.

Thus our aim was to deconstruct the complex 3D environment within an exemplary scaffold type (Swiss roll [12,13]) and develop mini-bioreactors to simulate a well-defined aspect of the ‘Swiss-rolls’ interior architecture (i.e. open pore structure). This reductionist approach allowed us to investigate the interactions of different cell types with defined pore features *in situ* during long-term static or perfusion culture using various non-invasive microscopic techniques.

We have shown that two approaches were used by the cells in cortical astrocyte cultures to either ‘cover’ the pores without migrating inside (Approach I) or ‘bridge’ the pores by migrating inside whilst attaching onto the edge (Approach II) as illustrated in Fig. 3A.

In cultures of cortical astrocytes the interactions of cells with the pores could be classified to happen in three distinctive stages:

Stage I: Individual oligodendrocyte precursor cells or type 2 astrocytes partly ‘covered’ or ‘bridged’ the pores using approaches I and II, but all these cells died off eventually.

Stage II: Type 1-astrocyte colonies gradually dominated the culture and ‘covered’ some of the pores. However, before the cultures were confluent, the astrocyte sheets that partly covered the pores were always pulled away from the pores, or the freely suspended cell membrane was “torn apart” by the apparent pull of other astrocytes within the same colonies, but on non-porous areas, that migrated away from the pores.

Stage III: When the cultures were confluent, astrocytes migrated into the pores, formed circular membranes with very regular and apparently tensed structures, and seemed to pull other astrocytes into the pores.

Fibroblasts interacted with the pores in a way that was very different from that observed from cortical astrocyte cultures. First of all, fibroblasts mainly ‘bridged’ the pores after migrating inside these (approach II). Secondly, the fibroblasts were quicker to seal the pores, which was similar to previous observations using human dermal fibroblasts [20,21] and 3T3 fibroblasts [15]. Thirdly, fibroblasts were quicker to migrate through the pores onto the other side of the substrate. Finally, although the bridging behavior of both types of cells was influenced by surface curvature, fibroblasts demonstrated the ability to bridge larger pores than astrocytes within a defined culture period. All these differences are probably due to the distinctive cell–cell and cell–substrate interactions, and different migration capabilities, as individual fibroblasts were able to migrate freely, whilst astrocytes often stayed within tight colonies.

One similarity between astrocytes and fibroblasts was that all the freely suspended multicellular structures that formed within the pores were robust. The structures either cell type formed were very resilient to medium perfusion. The formation of freely suspended cell-bridges was not obviously influenced, or even prevented by medium perfusion within the flow regime investigated. This cellular resilience was a surprise to us as perfusion was initially expected to be an efficient strategy to prevent the pores from being sealed by the ‘fragile’ cells. Ideally, some of the pores in 3D scaffold transplanted *in vivo* should remain open to allow nutrient diffusion as the neo-tissue forms [15]. Although medium perfusion was not sufficient to keep the pores open, our observations suggested that if the pore size was larger than 150–200 μm in diameter, other ‘soft’ approaches such as optimal cell seeding density together with culture time of less than 3–4 weeks might be sufficient to keep most of the pores open at least in the context of cortical astrocytes. Topographic features could also be incorporated at the stage of construct fabrication that might reduce pore closure: a wide range of cell types including astrocytes will align to micro-structured surfaces [31] and regular arrays of nano-pillars can provide surfaces with low adhesive properties [32,33]. Thus micro-grooves could be used to side-track the astrocytes from the pores, or regular arrays of nano-pillars could also be introduced to render the areas around the pores less adhesive, specifically for astrocytes.

Fibroblasts are common cells in many tissues and are usually involved in various wound healing processes where they migrate into a wound, surround and even invade the grafted tissues [24,25]. Due to their ability to quickly ‘bridge’ the pores, and to commute between different sides of the porous substrates through the pores, the open pores in the envisaged Swiss rolls might open the doors for fibroblast invasion. This potentially could influence the performance of the final engineered scaffold. Thus, it might be necessary to block the invasion of fibroblasts into the ‘Swiss-rolls’ for specific applications such as vascular tissue engineering. Apparently, large pore size and culture time period are not efficient to prevent fibroblasts invasion. However, chemical strategies such as coating the outer surface using non-adhesive materials to prevent fibroblasts adhesion, topographic strategies such as micro-grooves to side-track fibroblasts from the pores or nano-pillars to make the areas around the pores less adhesive for fibroblasts might be worth trying at the stage of scaffold design and fabrication.

The complex interactions of different cell types especially fibroblasts with defined pore features have been previously investigated using silicon nitride [15], electro-spun fibers [20] and nickel grids [21]. Apart from suspending the porous substrate in the medium to avoid the influence of tissue culture surface on cell–pore interactions [15,20,21], the methodologies developed in this paper differ from previous investigations in three ways: First of all, the combination of two static culture modules enabled us to observe the complex 3D cell structures within the pores from different angles, analyse and compare the abilities of different cell types to invade porous scaffolds. Secondly, the perfusion culture module made it possible for us to investigate the cell–pore interactions in perfusion culture. Thirdly, we used a new microscopic technology SICM [28] to demonstrate the morphological and topographical features of various cell structures during culture with high resolution (<0.1 μm) [28]. Due to these new developments, we are the first group to systematically investigate the interactions of various cells in cortical astrocyte cultures with define pore features. Apart from confirming the previous observation of fibroblasts’ joint efforts to bridge the pores [15], our research demonstrated that fibroblasts have the ability to bridge large pores (100–350 μm), and even up to 3 mm in diameter if sufficient time was provided, which is closer to the real in tissue situation. We believe that the methodologies developed in this research such as combining a thin layer of porous PDMS membrane with the miniaturized static and perfusion culture modules integrated with various non-invasive microscopes could also be used to systematically investigate the interactions of different cell types with defined pore feature before a specific engineered scaffold is fabricated and used for tissue engineering.

## 5 Conclusions

This research demonstrated that the two different cell types investigated showed time and density dependent variant pore sealing behaviors. These were influenced by pore size, initial cell density and culture period, but not obviously by medium perfusion within the range of shear forces investigated. Fibroblasts bridge pores given time - of almost any size, interact with the inner aspect of the pore and migrate across from one side of the membrane to the other. Astrocytes interact with the pores and bridge these as well, but they need to be almost confluent on the areas surrounding the pore before they start to seal pores smaller than about 100 μm as a sheet, that closes iris-like. These findings are important basic data about the usability of pores to keep mass transport routes open within scaffolds that could inform the design and fabrication of suitable scaffolds for various applications in tissue engineering. The methodology developed here, specifically the combining of a thin layer of a porous polymer membrane with the miniaturized static and perfusion culture modules that are accessible to various non-invasive microscopies could be further used to systematically investigate the interactions of other relevant cell types with defined pore features before a tissue specific scaffold is fabricated and thus speed up the engineering of designed scaffolds for tissue engineering.

## Figures and Tables

**Figure:**
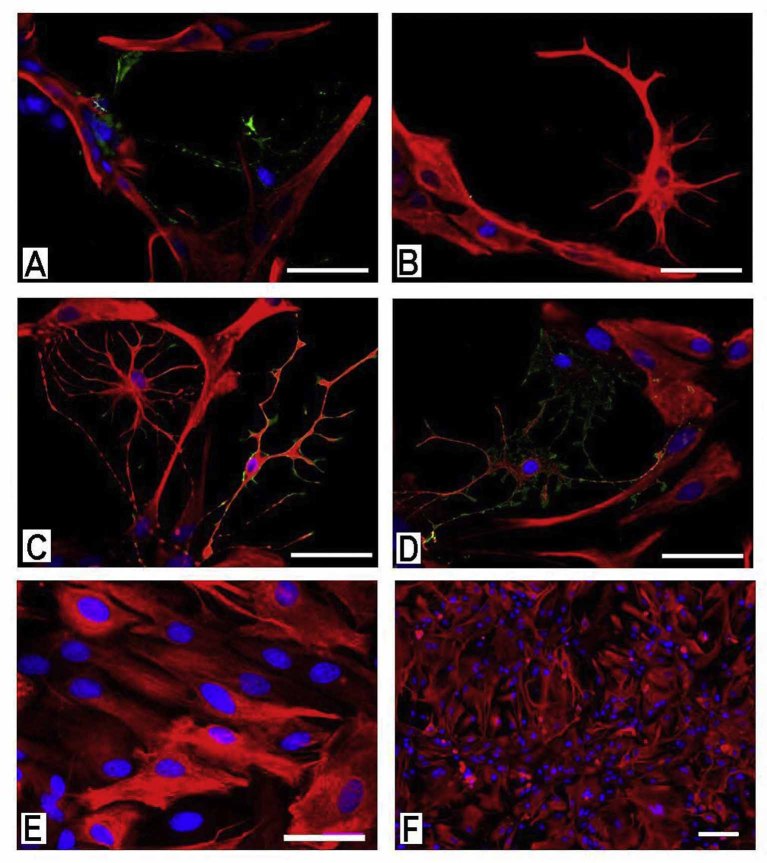
Supplementary Figure 1: Epifluorescence micrographs of cells in cortical astrocyte cultures immuno-labelled with (A and B): anti-GFAP protein (Red) and the O4 antibody (Green), (C and D) anti-GFAP protein (Red) and the A2B5 antibody (Green) after 1 day in culture, and (E and F) with anti-GFAP protein (Red) after two weeks in culture and have become confluent on glass cover slips. The nuclei were all visualized with DAPI (blue). Bar = 100 μm.

**Figure:**
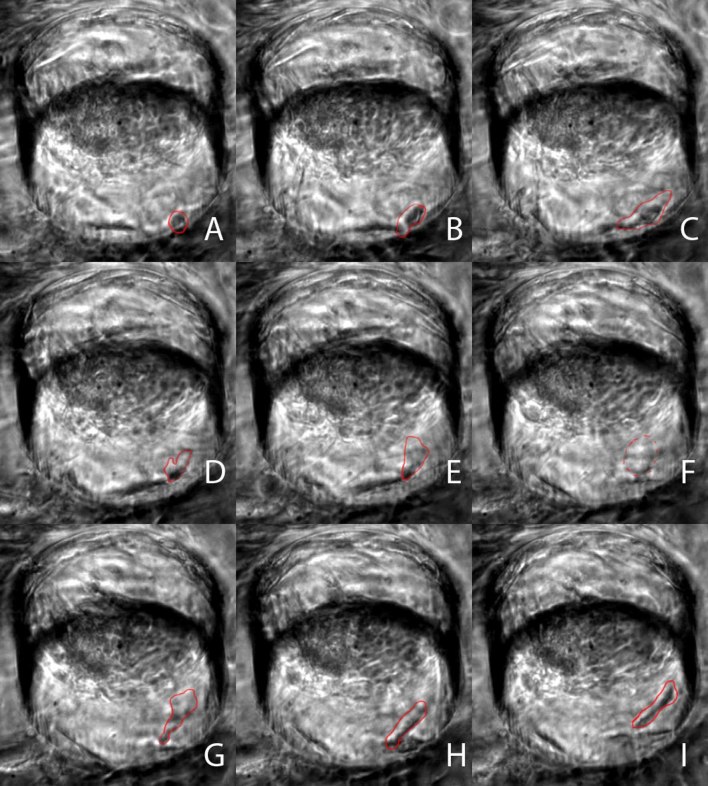
Supplementary Figure M7 Montage: Montage of the time-lapse video to be seen in Supplementary Figure 6, of fibroblasts on porous PDMS membrane that has been suspended tilted (static culture module II) after culture 14 days. In this montage one of the cells that cross from below the pore to the other side as seen in Figure M5 is outlined and highlighted in red.

**Table 1:** Medium flow and shear rates used in the perfusion experiments.

Flow rate [ml/h]	80	160	240	320	400
Shear rate [1/s]	1.1	2.2	3.3	4.4	5.6

## Appendix

Figures with essential color discrimination. Figs. 3, 5–7 in this article are difficult to interpret in black and white. The full color images can be found in the online version, at doi:10.1016/j.biomaterials.2010.11.046.

## Appendix Supplementary material

Supplementary Figure 2: Time-lapse video of type 1 astrocytes on porous PDMS membrane in static culture module I after culture 4 days the movement of the cells within the pores and in the surrounding indicate that the cells within the pores were pulled out by the cells on the non-porous area. 20x mag; images taken at 6 min/frame, and displayed at 25 frames/s.

Supplementary Figure 3: Time-lapse video of type 1 astrocytes on porous PDMS membrane in static culture module I after culture 35 days the movement of the cells within the pores and in the surrounding indicate that the cells within the pores were pulled in by the cells within the pore. 20x mag; images taken at 4 min/frame, and displayed at 25 frames/s.

Supplementary Figure 4: Time-lapse video of fibroblasts on porous PDMS membrane in perfused culture module at lower (left) and higher (right) density during pulsatile flow. The cells within the pores and the surrounding area are not affected by the flow. Even individual cells (left), whose thin extensions span the pore are not dislodged, ruptured or in any other way obviously affected by the flow regime. 32x mag; images taken at 1 min/frame, and displayed at 25 frames/s.

Supplementary Figure 5: Time-lapse video of astrocytes on porous PDMS membrane in perfused culture module at lower (left) and higher (right) density during pulsatile flow. The cells within the pores and the surrounding area are not affected by the flow. Even individual cells (left), whose thin extensions span the pore are not dislodged, ruptured or in any other way obviously affected by the flow regime. 32x mag; images taken at 1 min/frame, and displayed at 25 frames/s.

Supplementary Figure 6: Time-lapse video of fibroblasts on porous PDMS membrane on a tilted membrane (static culture module II) after culture 14 days the movie shows that fibroblasts migrate across the pore from either side to the respective other. 32x mag; images taken at 5 min/frame, and displayed at 25 frames/s.

Supplementary Figure 8: Time-lapse video of astrocytes on porous PDMS membrane on a tilted membrane (static culture module II) the movie shows that astrocytes although entering, and interacting with the inner aspect of the pore do rarely migrate across these. 32x mag; images taken at 5 min/frame, and displayed at 25 frames/s.
