# Supplementary figures and images for: The interactions of astrocytes and fibroblasts with defined pore structures in static and perfusion cultures
Source: Biomaterials. 2011 Mar;32(8):2021–31. doi: 10.1016/j.biomaterials.2010.11.046 (PMC3440599; doi:10.1016/j.biomaterials.2010.11.046)

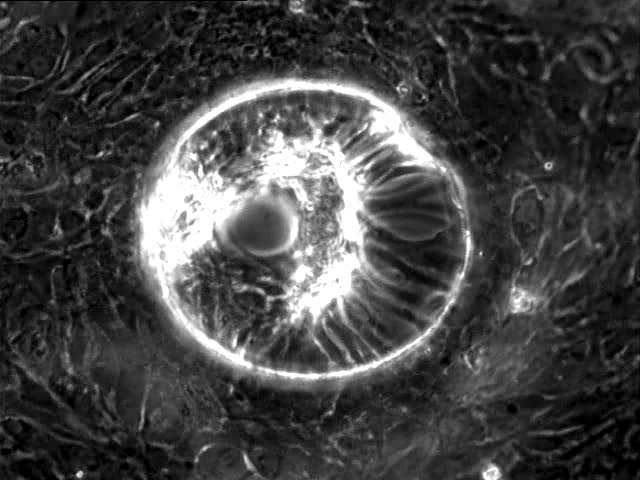

Supplement: Supplementary file 2 — Supplementary Figure 3: Time-lapse video of type 1 astrocytes on porous PDMS membrane in static culture module I after culture 35 days the movement of the cells within the pores and in the surrounding indicate that the cells within the pores were pulled in by the cells within the pore. 20x mag; images taken at 4 min/frame, and displayed at 25 frames/s. [file mmc2.jpg]

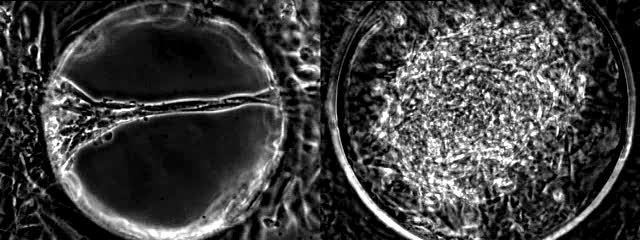

Supplement: Supplementary file 3 — Supplementary Figure 4: Time-lapse video of fibroblasts on porous PDMS membrane in perfused culture module at lower (left) and higher (right) density during pulsatile flow. The cells within the pores and the surrounding area are not affected by the flow. Even individual cells (left), whose thin extensions span the pore are not dislodged, ruptured or in any other way obviously affected by the flow regime. 32x mag; images taken at 1 min/frame, and displayed at 25 frames/s. [file mmc3.jpg]

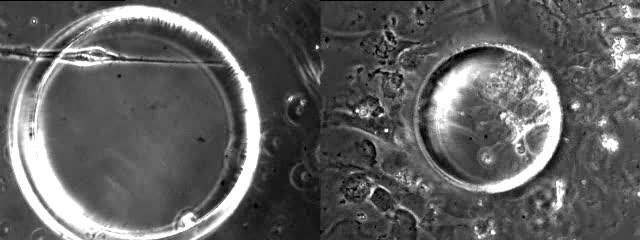

Supplement: Supplementary file 4 — Supplementary Figure 5: Time-lapse video of astrocytes on porous PDMS membrane in perfused culture module at lower (left) and higher (right) density during pulsatile flow. The cells within the pores and the surrounding area are not affected by the flow. Even individual cells (left), whose thin extensions span the pore are not dislodged, ruptured or in any other way obviously affected by the flow regime. 32x mag; images taken at 1 min/frame, and displayed at 25 frames/s. [file mmc4.jpg]

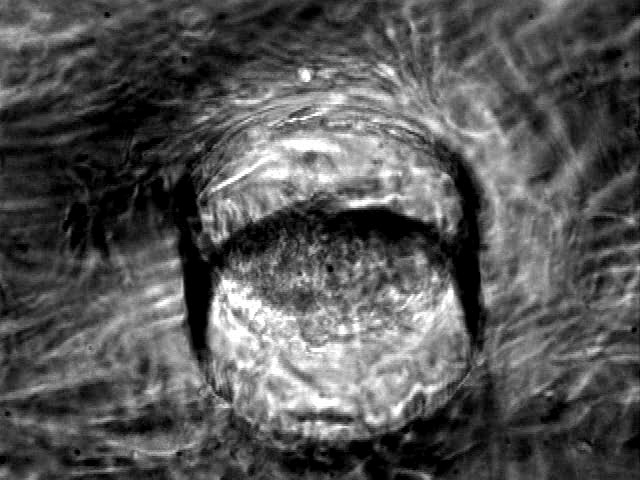

Supplement: Supplementary file 5 — Supplementary Figure 6: Time-lapse video of fibroblasts on porous PDMS membrane on a tilted membrane (static culture module II) after culture 14 days the movie shows that fibroblasts migrate across the pore from either side to the respective other. 32x mag; images taken at 5 min/frame, and displayed at 25 frames/s. [file mmc5.jpg]
